# A Radiomic-Based Machine Learning System to Diagnose Age-Related Macular Degeneration from Ultra-Widefield Fundus Retinography

**DOI:** 10.3390/diagnostics13182965

**Published:** 2023-09-15

**Authors:** Matteo Interlenghi, Giancarlo Sborgia, Alessandro Venturi, Rodolfo Sardone, Valentina Pastore, Giacomo Boscia, Luca Landini, Giacomo Scotti, Alfredo Niro, Federico Moscara, Luca Bandi, Christian Salvatore, Isabella Castiglioni

**Affiliations:** 1 DeepTrace Technologies S.R.L., 20122 Milan, Italy; interlenghi@deeptracetech.com (M.I.); venturi@deeptracetech.com (A.V.); bandi@deeptracetech.com (L.B.); 2 Department of Medical Science, Neuroscience and Sense Organs, Eye Clinic, University of Bari Aldo Moro, 70121 Bari, Italy; gcsborgia@hotmail.it (G.S.); valentinapastore@hotmail.it (V.P.); bosciagiacomo@gmail.com (G.B.); lucalandi4@gmail.com (L.L.); giacomo.scotti@tiscali.it (G.S.); federico.moscara@gmail.com (F.M.); 3 National Institute of Gastroenterology—IRCCS “Saverio de Bellis”, 70013 Castellana Grotte, Italy; rodolfo.sardone@irccsdebellis.it; 4 Unit of Statistics and Epidemiology, Local Healthcare Authority of Taranto, 74121 Taranto, Italy; 5 Eye Clinic, Hospital “SS. Annunziata”, ASL Taranto, 74121 Taranto, Italy; alfred.nir@tiscali.it; 6 Department of Science, Technology and Society, University School for Advanced Studies IUSS Pavia, 27100 Pavia, Italy; 7 Department of Physics “Giuseppe Occhialini”, University of Milan-Bicocca, 20126 Milan, Italy; isabella.castiglioni@unimib.it

**Keywords:** age-related macular degeneration (AMD), ultra-widefield (UWF), fundus retinography (FRT), artificial intelligence (AI), machine learning (ML), radiomics, deep learning (DL), detection, image segmentation, explainability

## Abstract

The present study was conducted to investigate the potential of radiomics to develop an explainable AI-based system to be applied to ultra-widefield fundus retinographies (UWF-FRTs) with the objective of predicting the presence of the early signs of Age-related Macular Degeneration (AMD) and stratifying subjects with low- versus high-risk of AMD. The ultimate aim was to provide clinicians with an automatic classifier and a signature of objective quantitative image biomarkers of AMD. The use of Machine Learning (ML) and radiomics was based on intensity and texture analysis in the macular region, detected by a Deep Learning (DL)-based macular detector. Two-hundred and twenty six UWF-FRTs were retrospectively collected from two centres and manually annotated to train and test the algorithms. Notably, the combination of the ML-based radiomics model and the DL-based macular detector reported 93% sensitivity and 74% specificity when applied to the data of the centre used for external testing, capturing explainable features associated with drusen or pigmentary abnormalities. In comparison to the human operator’s annotations, the system yielded a 0.79 Cohen *κ*, demonstrating substantial concordance. To our knowledge, these results are the first provided by a radiomic approach for AMD supporting the suitability of an explainable feature extraction method combined with ML for UWF-FRT.

## 1. Introduction

Age-related Macular Degeneration (AMD) is a leading cause of irreversible vision loss among individuals older than 55, with 200,000 new diagnoses per year in the United States [1]. The neovascular form of AMD (nAMD) originates from the development of an exudative macular neovascularisation (MNV), which may result in a severe impairment of the central vision due to the continuous damage to photoreceptors and retinal pigment epithelium (RPE) [2,3,4,5].

At present, several therapies have been proposed to inhibit the MNV-related exudation [6,7]. Specifically, in the last years, intravitreal injections of anti-vascular endothelial growth factor (anti-VEGF) became the first line approach to deal with this condition, revolutionising the care of affected people [8,9,10]. Indeed, such disease is treatable with good outcomes if detected early. 

Despite that, AMD is a degenerative, progressive disease and the symptoms become worse over its development. Dry AMD does not show any symptoms in the early stage; for some patients, mild symptoms show in the intermediate stage, including blurriness in the central vision or reduced vision in low lighting. Late stage, both for dry and wet AMD, show more severe and frequent symptoms among the affected patients, including greater blurriness in the central vision, blank spots, dull colours, or the perception of straight lines as crooked or wavy. Since the symptoms tend to appear and become more severe in the late stage of the disease, early diagnosis for AMD is very difficult. Moreover, accurate diagnosis implies high costs for national health systems and rely on examinations performed by expert operators able to identify the early stage of the illness. The clinical diagnosis of AMD is commonly made through fundus retinography (FRT). However, conventional FRT has a limited field of view (30 to 50 degrees) [11] and requires many acquisitions to compose an adequate portion of the fundus. This limitation has been overcome in recent years, thanks to the novel technology ultra-widefield FRT (UWF-FRT), which is able to capture, with a single shot, an angular region up to 220° [12].

The parallel development of new and advanced imaging techniques and of Artificial Intelligence (AI) methods, the state-of-the-art of many image computational tasks, such as imaging processing and recognition, has dramatically improved. Indeed, AI refers to “development of computer systems able to perform tasks by mimicking human intelligence, such as visual perception, decision making, and voice recognition” [13]. Thus, AI application in a hyper specialised field of medicine such as ophthalmology, and in particular in the recognition of retinal conditions from RT, became a reliable approach.

In AMD, RT allows readers to detect specific retinal signs of degenerations, such as the presence of drusen, neovascularisations, fibrovascular scars, and intraretinal or subretinal macular fluids.

The automatic detection and quantification of such image features can represent an efficient and objective assessment tool for the screening, early diagnosis, and, consequently, the care and the prognosis of the latter disease [14]. In this context, several Deep Learning (DL) models, such as Convolutional Neural Networks (CNNs), have been developed, achieving excellent performances. However, the DL models are considered to have limited explainability and limited biological interpretability (indeed, they are usually called “black-box” systems) since the features used for classification are extracted by the model itself, without any constraint for the features to be bound to relevant biomarkers that humans can address in the medical images. Many approaches have been proposed to solve the explainability issues of DL systems; such approaches can be categorised into three main types: visualisation methods, distillation methods, and intrinsic methods [15]. Visualisation methods focus on highlighting the regions of the image that provide the greatest contributions to the model’s predictions, yet without providing explanations of why such regions are relevant. Distillation methods are based on the creation of a secondary machine learning model that is trained to reproduce the output of the DL model; however, such methods can produce just hypotheses of how the DL model works [15]. Finally, intrinsic methods generate models that provide the explanation of the output together with the prediction. However, these methods toned to be trained on such explanations, thus requiring each training image to have a corresponding explanation labelled by an expert. Moreover, such methods are limited to providing case-by-case explanations and do not provide an overview of the model’s functioning. Finally, since such explanations are predicted by the model, they can contain mistakes and deceive the reader. In addition to the limitations of the explainability techniques proposed in the literature, DL models usually extract hundreds or even thousands of features. As an example, some of the most used CNNs are ResNet50 [16] and DenseNet201 [17], which extract 2048 and 1000 features, respectively, thus making it very difficult to interpret the model’s predictions, even when understanding the meaning of each feature. However, since many DL systems with good performances have been proposed in recent years for the AMD identification task, they are addressed in the Discussion section. 

Radiomics is a well-known image analysis technique that allows the extraction of quantitative descriptors from a digital radiological image, characterising a region or volume of interest in terms of morphology, intensity, and texture. Such characteristics correlate with the pathophysiology of many diseases, as proven in several cases where classification models to automatically classify the presence or absence of diseases (“Positive” vs. “Negative” task) were developed [18,19].

The objective of the present work is to investigate the potential of radiomics to develop and test an explainable AI-based system applied to RT for predicting the presence of the early signs of AMD within the macula and stratifying low- versus high-risk of AMD. The final goal was to provide clinicians with an automatic classifier and a signature of objective quantitative image biomarkers of the AMD. An ML-based radiomics model was developed for such a purpose based on intensity and texture analysis in the macular region, which was automatically detected by a DL-based macular detector.

## 2. Materials and Methods

### 2.1. Image Sets

To begin, 296 UWF-FRT of single eyes were retrospectively collected from subjects at risk of AMD. The study was approved by the Ethics Committee “Comitato etico IRCCS—Istituto tumori Giovanni Paolo II Bari”. Approval Code: 68/CE De Bellis. Approval Date: 9 April 2019. Informed consent about the diagnostics and clinical assessment was signed by each study participant. 

An image set of 145 UWF-FRT of single eyes from 102 subjects was used for the training, validation, and internal testing of a DL-based macular detector and an ML-based radiomics model. Among these UWF-FRT, 62 (43%) belonged to class “AMD” and 83 (57%) belonged to class “Negative”, according to clinical evaluation. Such images were gathered from the “Salus in Apulia Study”, a population-based prospective cohort comprising subjects, residents in Castellana Grotte, Bari (Puglia Region, Southern Italy), over 65 years. All the images were taken using a Daytona scanner (Optos, Dunfermline, UK) by a group of ophthalmologists, who were experts in retinal diseases. The same group of ophthalmologists selected images of healthy retinas and images of retinas affected from AMD. 

Separately, the remaining 151 images of UWF-FRT of single eyes from 85 subjects were used to externally test both the DL-based macular detector and the ML-based radiomics model. Among these UWF-FRT, 108 (72%) belonged to class “AMD” and 43 (28%) belonged to class “Negative”, according to clinical evaluation. UWF-FRT for external testing were taken using another Daytona scanner from patients referred to the Policlinico of Bari retina service for IVT injections for neovascular-AMD. Since the asymmetricity of the disease between the two eyes, the same group of ophthalmologists cited before were able to select both patients with a healthy fellow eye of the one they were treated for, and patients with a fellow eye affected by non-neovascular-AMD.

### 2.2. DL-Based Macular Detector

A DL-based macular detector was developed to automatically identify the macula from the retinography image and to extract a circular region to be passed as input to the ML-based radiomics model. In order to provide a reference standard for such object detector, an operator created the region of interest (ROI) for each image using the Trace4Research segmentation tool (DeepTrace Technologies S.R.L., Milan, Italy). The macula centre was selected by the operator, a circular contour was automatically produced around it (250 pixels radius, ~2 mm) and subsequently adjusted by the operator in terms of position and radius. 

The detector used was a YOLO (You Only Look Once) V2 network [20], which exploits the features extracted from a ResNet50 [16], pre-trained on the ImageNet dataset [21], combining high-level and low-level features through a reorganisation layer. The detector was trained for 100 epochs on a single GPU. The predictions obtained from the trained detector are bounding boxes candidate to contain the ROI. For each image, the box predicted with the highest confidence score was selected, its centre was extracted, and the circle of radius 250 pixels around it, which represents the predicted ROI, was created.

The dataset was partitioned into training (56%), validation (14%), and internal testing (30%) sets. On the training set, the distributions of the x and y position of the macula were analysed, finding that the macula was always located around the centre of the UWF-FRT. The coordinates of the centres were (mean ± std): x = 1971 ± 46 and y = 1588 ± 52, while all the images had a resolution of 3900 × 3072 pixels. Starting from this observation, a range for cropping the UWF-FRT without excluding the macula was selected; the pixels in the range (901–2700) on the *x*-axis and (801–2600) on the *y*-axis were retained, thus obtaining a squared region of 1800 × 1800 pixels. These ranges, expressed as a percentage of the image size, correspond to [23%, 69%] and [26%, 85%], respectively. Moreover, the same ranges, expressed in standard deviations (σ) from the mean position of the closest borders of the ROIs, correspond to [18σ, 10σ] and [10σ, 15σ]. During the training phase, the ROIs segmented by the operator were preprocessed, halving the radius, and the images were resampled to a target size of 300 × 300 pixels, adjusting the ROIs accordingly.

The detector’s performance was tested comparing its predictions with the human operator’s manual ROI on the Dice Similarity Coefficient (DSC) [22], defined by Equation (1):

(1) $$DSC=2\frac{\left|A\cap B\right|}{\left|A\right|+\left|B\right|}$$

where $\left|A\right|$ and $\left|B\right|$ are the cardinalities, i.e., the count of pixels, of the two ROIs, and $\left|A\cap B\right|$ is the cardinality of their intersection.

Furthermore, the detector was evaluated with the use of the ML-based radiomics model. The evaluation was carried out by comparing the latter’s performances in two different settings: the first using the human operator’s manual ROIs and the second using the detector’s predictions. The comparison was obtained by computing Cohen *κ* [23], defined by Equation (2):

(2) $$\kappa =\frac{p_{o}-p_{c}}{1-p_{c}}$$

where $p_{o}$ is the proportion of cases with classification’s agreement and $p_{c}$ is the proportion of cases in which classification’s agreement is expected by chance.

### 2.3. ML-Based Radiomics Model

Radiomic methodology was implemented in the present work, achieving adherence to the guidelines identified by the Image Biomarker Standardization Initiative (IBSI) [24] with regards to methodology, features definition, and features reporting. The workflow was developed using the Trace4Research platform [25].

Specifically, the radiomic workflow consisted of:

1. The manual segmentation of the ROI, performed by one expert operator, as described in the DL-based macular detector section.
2. The preprocessing of pixel intensities in the ROI, which included grayscale pixel conversion and resampling, using a down-sampling scheme of 2 to 1 pixels.
3. The computation of Radiomics features from the segmented ROI, belonging to different features families: Intensity-based Statistics, Intensity Histogram, Gray-Level Co-occurrence Matrix (GLCM), Gray-Level Run Length Matrix (GLRLM), Gray-Level Size Zone Matrix (GLSZM), Neighbourhood Gray Tone Difference Matrix (NGTDM), Neighbouring Gray Level Dependence Matrix (NGLDM). Their definition, computation, and nomenclature are reported in the IBSI guidelines [24]. Steps (2) and (3) were performed using the Trace4Research radiomics tool. It must be noted that Intensity Histogram features were computed after an intensity discretisation of the ROI, using a fixed number of 64 bins. Texture features (GLCM, GLRLM, GLSZM, NGTDM, NGLDM) were computed after an intensity discretisation of the ROI, using a fixed number of 64 bins.
4. The selection of relevant features, addressing stability and repeatability with respect to different segmentations and test-retest study. This was evaluated by computing ICC (1,1) [26] (ICC > 0.80) and statistically comparing features obtained by data augmentation strategies. Data augmentation comprised random rotation of the original image and the segmented ROI and random manipulation of the segmented ROI.
5. The training, validation, and internal testing of two supervised machine-learning models, using a nested 10-fold cross-validation approach. The first model’s architecture was an ensemble of three ensemble models, each composed of 100 random-forest classifiers combined with Gini index and majority-vote rule. The second model’s architecture was an ensemble of three ensemble models, each composed of 100 support vector machines (SVM) combined with principal components analysis, Fisher Discriminant Ratio, and majority-vote rule. The minority class (“AMD”) was oversampled by adaptive synthetic sampling method (ADASYN) [27]. The performances of the two models were measured in terms of mean Accuracy, Sensitivity, Specificity, Positive Predictive Value (PPV), Negative Predictive Value (NPV) (defined by Equations (3)–(7)), Area Under the Receiver Operating Characteristic Curve (ROC-AUC) [28], and also by evaluating the 95% confidence intervals (CI). Among the two models, the one with the highest ROC-AUC was chosen as the best classification model.

Given that “AMD” represents the positive class and that TP=True Positives, TN=True Negatives, FP=False Positives, and FN=False Negatives, the following metrics are defined:

(3) $$Accuracy=\frac{TP+TN}{FP+FN}$$

(4) $$Sensitivity=\frac{TP}{TP+FN}$$

(5) $$Specificity=\frac{TN}{TN+FP}$$

(6) $$PPV=\frac{TP}{TP+FP}$$

(7) $$NPV=\frac{TN}{TN+FN}$$

### 2.4. Statistical Analysis

Statistical analysis was carried out with the Trace4Research platform. 

The accuracy of the DL-based macular detector was calculated in terms of DICE coefficient. Diagnostic performances of the ML-based radiomics model were obtained in terms of sensitivity, specificity, and accuracy.

The distribution of the most relevant features in the two classes was presented both numerically, by their medians with 95% CI, and graphically, by the use of violin and box plots for intuitive visualisation and interpretation. For each of such features, the statistical significance in discriminating the two classes was assessed by a non-parametric univariate Wilcoxon rank-sum test (Mann–Whitney U test) [29] with Bonferroni correction [30] and significance levels set at 0.05 and 0.005.

## 3. Results

### 3.1. DL-Based Macular Detector

**Figure 1:**
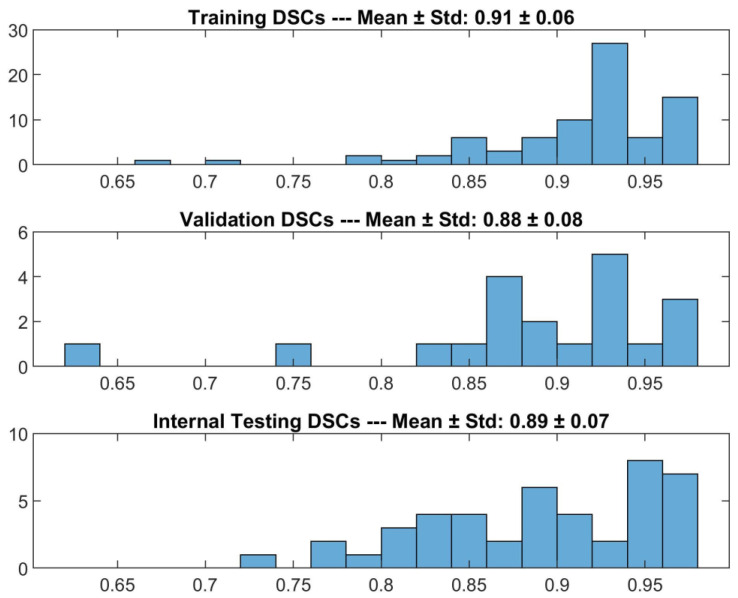
DSCs for the comparison between DL-based macular detector’s predictions and human operator’s manual ROIs, on the training, validation and internal testing sets.

The detector’s performance is reported in Figure 1. The detector was able to find the macula in 95% of the cases in the internal testing set. Specifically, when the detector failed to identify the macula, the prediction was replaced by the mean position of the human operator’s manual ROIs calculated on the training set. Overall, the detector achieved 0.89 ± 0.07 (mean ± std) DSC on the internal testing set. Furthermore, on the training and validation sets the detector identified the macula for all the cases, achieving 0.91 ± 0.06 and 0.88 ± 0.08 (mean ± std) DSC, respectively.

**Figure 2:**
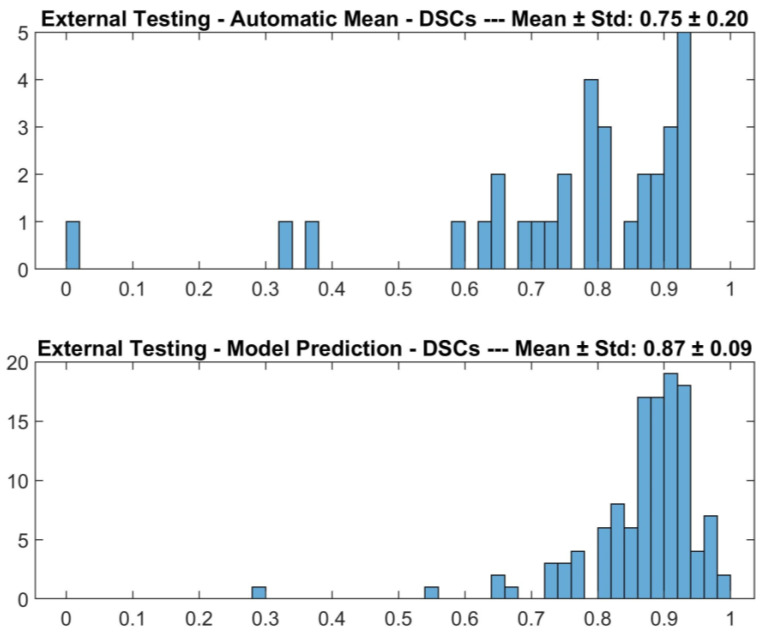
DSCs for the comparison between DL-based macular detector’s predictions and human operator’s manual ROIs, on the external testing set.

**Figure 3:**
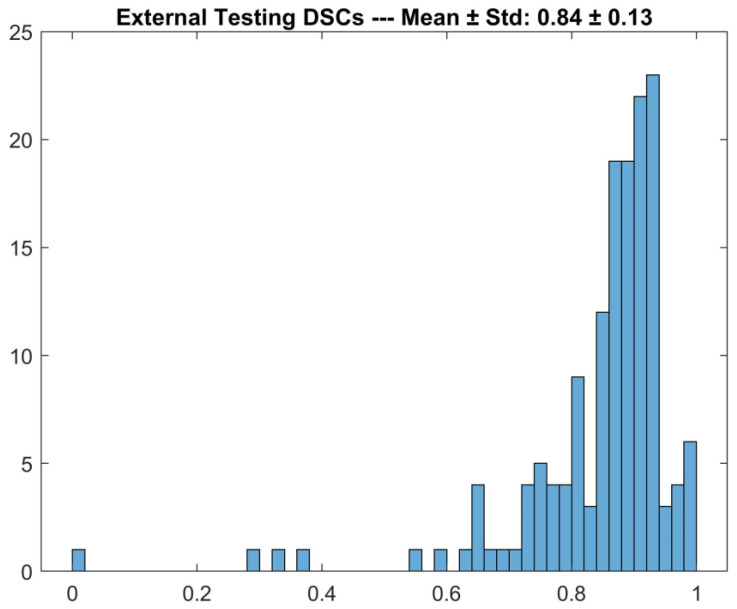
DSCs for the comparison between DL-based macular detector’s predictions and human operator’s manual ROIs, on the external testing, split according to whether the detector failed (above, Automatic Mean) or succeeded (below, Model Prediction) in detecting the macula.

As shown in Figure 2, the detector achieved 0.84 ± 0.13 (mean ± std) DSC on the external testing set. As shown in Figure 3, the detector failed to identify the macula in 31/151 cases (21%); in such cases, the mean position calculated on the training set was used, achieving 0.75 ± 0.20 (mean ± std) DSC.

**Figure 4:**
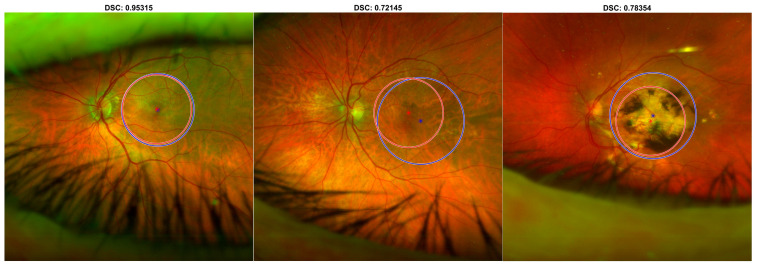
Predictions of the DL-based macular detector, on representative UWF-FRT from the internal testing set. The predictions (in red) are compared with the reference standard human operator’s manual ROIs (in blue).

**Figure 5:**
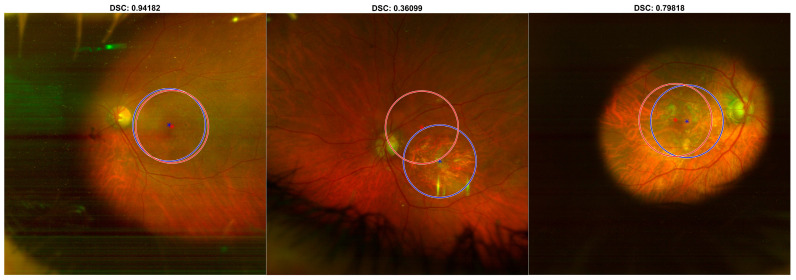
Predictions of the DL-based macular detector, on representative UWF-FRT from the external testing set. The predictions (in red) are compared with the reference standard human operator’s manual ROIs (in blue).

In Figure 4 and Figure 5, three examples of predictions are presented on UWF-FRT from the internal testing set and the external testing set, respectively.

### 3.2. Radiomic-Based ML Model

From each segmented ROI considered in this study, IBSI-compliant radiomic features were computed, for a total of 270 features. For the classification task of interest (62 images from class “AMD” vs. 83 images from class “Negative”), these features were used for training, cross-validation, and internal testing (nested 10-fold cross-validation) of the two different models of three ensembles of ML classifiers considered in this work (random forest and SVM). 

**Figure 6:**
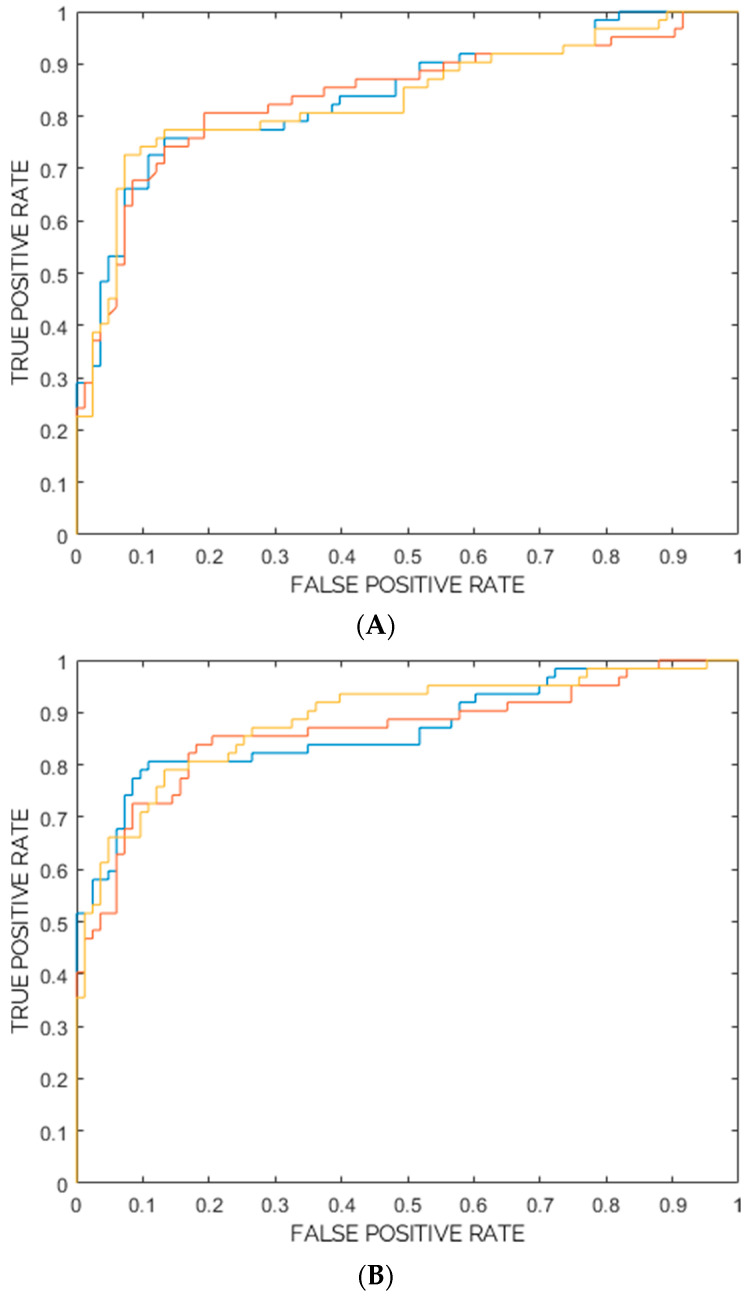
(**A**) ROC Curve for each of the three ensembles (represented in blue, red and yellow) of random forest classifiers (from Internal Testing). (**B**) ROC Curve for each of the three ensembles (represented in blue, red and yellow) of support vector machine classifiers (from Internal Testing).

Table 1 show ROC-AUC, Accuracy, Sensitivity, Specificity, PPV, and NPV as obtained from the training, cross-validation, and internal testing of the two models. Furthermore, for each model, ROC curves for the three ensembles are plotted in Figure 6A,B. Based on ROC-AUC, the model composed of SVM classifiers showed the best ROC-AUC and was therefore chosen as the best model for the task of interest (62 images from class “AMD” vs. 83 images from class “Negative”).

Images from the second centre (external testing dataset) were used to evaluate both the ML-based radiomics model and the DL-based macular detector, measuring the performances of the first comparing human operator’s manual ROIs and DL-based macular detector’s predictions. In Table 2 the external testing performances are reported in terms of sensitivity, specificity, PPV, and NPV, with a corresponding 95% CI and statistical significance. Finally, the reliability analysis resulted in an overall linear-weighted Cohen *κ* equal to 0.79 (95% CI of 0.66–0.89).

### 3.3. Statistical Analysis

**Figure 7:**
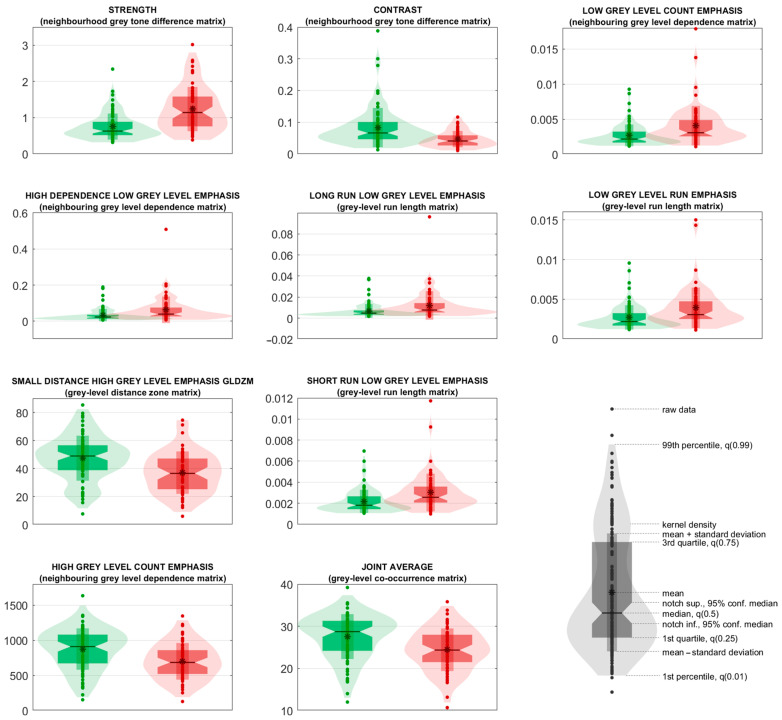
Violin and box plots for the top 10 radiomic predictors. Violin and box plots of “AMD” and “Negative” classes are in red and green, respectively.

The 10 top radiomic predictors are listed in Table 3, with their feature family and nomenclature, as reported in IBSI guidelines, and with the corresponding median values, 95% Cis, and the results from univariate statistical sum rank tests with adjusted *p*-values. Predictors are ranked according to their statistical significance and their frequencies in the classifiers. The violin plot and boxplot of the 10 top radiomic predictors are shown in Figure 7. The explanation of what such features represent and their alignment with the elements typically observed by clinicians in the images are addressed in the Discussion section.

### 3.4. Classification Result

**Figure 8:**
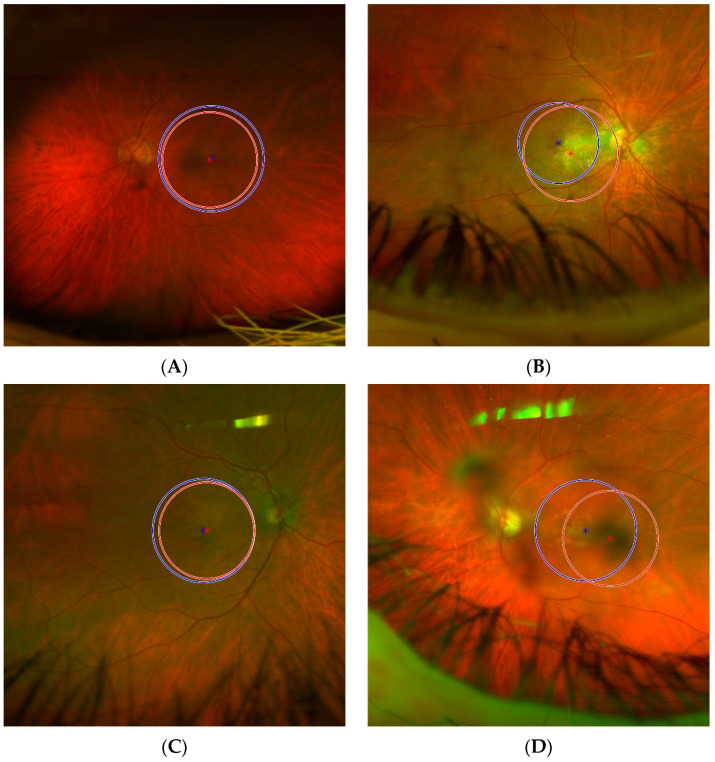
Representative examples from external testing, one example for each classification outcome. The ML-based radiomics model’s predictions are obtained from ROIs obtained by the DL-based macular detector (in red). For each ROI it is also reported the reference standard human operator’s manual ROIs (in blue). (**A**) Example of *True Positive* (“AMD” correctly classified as “AMD”). (**B**) Example of *True Negative* (“Negative” correctly classified as “Negative”). (**C**) Example of *False Positive* (“Negative” incorrectly classified as “AMD”). (**D**) Example of *False Negative* (“AMD” incorrectly classified as “Negative”).

Representative examples of classification outcomes from automatically detected ROIs in the external testing set are reported in Figure 8:

(A) In Figure 8A, an example of a *True Positive*, i.e., “AMD” classified as “AMD”, is shown. It is noteworthy that none of the 10 top predictors (reported in Table 3) excessively deviates from the expected distribution for “AMD” class (such distributions are graphically reported in Figure 7 and are computed excluding the external testing set). The ROI obtained from the DL-based automatic detector is characterised by the following feature values (percentile/difference with the mean in standard deviations units):
  1. Strength: 0.973489 (40°/−0.4σ)
  2. Contrast: 0.071115 (82°/0.9σ)
  3. Low Grey Level Count Emphasis: 0.00284707 (40°/−0.4σ)
  4. High Dependence Low Grey Level Emphasis: 0.025812 (25°/−0.5σ)
  5. Long Run Low Grey Level Emphasis: 0.00564284 (28°/−0.5σ)
  6. Low Grey Level Run Emphasis: 0.00285426 (45°/−0.4σ)
  7. Small Distance High Grey Level Emphasis: 34.7584 (44°/−0.2σ)
  8. Short Run Low Grey Level Emphasis: 0.00241731 (46°/−0.3σ)
  9. High Grey Level Count Emphasis: 751.594 (58°/0.2σ)
  10. Joint Average: 25.3098 (55°/0.2σ)
(B) In Figure 8B, an example of a *True Negative*, i.e., “Negative” classified as “Negative”, is shown. As with the previous case, in Figure 8A, all the 10 top predictors have reasonable values considering the expected distribution for “Negative” class. The values (percentile/standard deviations from the mean) are:
  1. Strength: 0.578356 (37°/−0.5σ)
  2. Contrast: 0.0518532 (31°/−0.5σ)
  3. Low Grey Level Count Emphasis: 0.0014604 (10°/−0.8σ)
  4. High Dependence Low Grey Level Emphasis: 0.00893454 (6°/−0.7σ)
  5. Long Run Low Grey Level Emphasis: 0.00237348 (5°/−0.7σ)
  6. Low Grey Level Run Emphasis: 0.00148348 (12°/−0.8σ)
  7. Small Distance High Grey Level Emphasis: 46.9358 (48°/0.0σ)
  8. Short Run Low Grey Level Emphasis: 0.00132314 (14°/−0.8σ)
  9. High Grey Level Count Emphasis: 1062.12 (71°/0.6σ)
  10. Joint Average: 31.6357 (80°/0.8σ)
(C) In Figure 8C, an example of a *False Positive*, i.e., “Negative” classified as “AMD”, is shown. Almost all the 10 top predictors have values that greatly deviate from the expected distribution for “Negative” class, with five predictors assuming values over the 100° percentile, i.e., values outside of the range the predictors had in the training set. This explains why the classification model is deceived and its prediction is wrong. The values (percentile/standard deviations from the mean) are:
  1. Strength: 2.80782 (>100°/5.7σ)
  2. Contrast: 0.0186873 (1°/−1.0σ)
  3. Low Grey Level Count Emphasis: 0.0103925 (>100°/5.0σ)
  4. High Dependence Low Grey Level Emphasis: 0.224731 (>100°/5.6σ)
  5. Long Run Low Grey Level Emphasis: 0.0419334 (>100°/5.3σ)
  6. Low Grey Level Run Emphasis: 0.00976493 (>100°/4.5σ)
  7. Small Distance High Grey Level Emphasis: 17.2006 (3°/−1.9σ)
  8. Short Run Low Grey Level Emphasis: 0.00681908 (99°/4.2σ)
  9. High Grey Level Count Emphasis: 343.866 (6°/−1.8σ)
  10. Joint Average: 16.7826 (3°/−2.0σ)
(D) In Figure 8D, an example of a *False Negative*, i.e., “AMD” classified as “Negative”, is shown. The first ranked predictor (strength) has a value under the 1° percentile and some more predictors have values at the boundaries of the distribution expected for the “AMD” class. Once again, these outlier values explain why the prediction is wrong. The values (percentile/standard deviations from the mean) are:
  1. Strength: 0.376468 (<1°/−1.4σ)
  2. Contrast: 0.0840939 (89°/1.5σ)
  3. Low Grey Level Count Emphasis: 0.00187011 (12°/−0.8σ)
  4. High Dependence Low Grey Level Emphasis: 0.00931938 (3°/−0.7σ)
  5. Long Run Low Grey Level Emphasis: 0.00277563 (7°/−0.7σ)
  6. Low Grey Level Run Emphasis: 0.00189526 (13°/−0.8σ)
  7. Small Distance High Grey Level Emphasis: 26.2522 (28°/−0.7σ)
  8. Short Run Low Grey Level Emphasis: 0.00172662 (17°/−0.7σ)
  9. High Grey Level Count Emphasis: 851.997 (70°/0.6σ)
  10. Joint Average: 28.0738 (75°/0.7σ)

## 4. Discussion

Early AMD detection is crucial in preventing irreversible vision loss in people suffering from the disease and to ameliorate their quality of life. 

Angular limitation of FRT as a diagnostic tool for AMD applied to the macula region has been overcome by UWF-FRT, which is able to measure a larger region.

AI algorithms may improve UWF-FRT image processing in the AMD population and be more cost-effective than employee operators. Compared to the clinicians’ efforts, the use of AI results in an extensive reduction in the manpower required for extracting knowledge from UWF-FRT. This takes advantage of AI algorithms’ abilities to extrapolate features from the images at different levels of autonomy, reproducibility, and explainability.

In this work, a fully automatic, highly explainable, and reproducible AI algorithm was developed that was able to evaluate the presence of the early signs of AMD within the subject macula by means of capturing specific local quantitative signs of the disease in the UWF-FRT. 

The proposed AI algorithm is composed of a DL-based macular detector, which automatically detects and locates the macula on UWF-FRT defining the ROI, and an ML-based radiomics model, which extracts the relevant quantitative features from the ROI and classifies the subject as with AMD or negative.

For this purpose, 145 UWF-FRT cases were retrospectively collected from one centre and clinically evaluated to train, validate, and internally test the two AI models. Moreover, an additional 151 UWF-FRT cases were collected retrospectively from a second centre and clinically evaluated to test both the AI models externally.

When tested on the human operator’s manual ROIs, the ML-based radiomics classifier achieved mean sensitivity and specificity of 77% and 87%, respectively, for the internal testing set, while both metrics reached 95% on the external testing set. When tested on the ROIs automatically obtained by the DL-based macular detector, the classifier showed mean sensitivity and specificity of 93% and 74%, respectively, suggesting that the use of the DL-based macular detector affects the performances of the algorithms on specificity rather than on sensitivity. However, the Cohen *κ* of 0.79 indicated good reproducibility and it is expected that the model could improve both sensitivity and specificity when trained on a larger number of UWF-FRT.

The proposed system presents important advantages, potentially setting the workload to zero when compared to the human reader’s clinical evaluation based on the observation of the macula region to detect disease features, thus automating the whole evaluation process and making the diagnosis independent from the reader. Furthermore, differently from the other works, our proposed tool has a good potential to discriminate any stage of AMD vs. negative.

To date, some authors have focused on the development of DL models able to complement the operators’ activity by combining human reading and AI [31,32,33,34]. Algorithms for AMD identification have been proposed using CNNs. For instance, on colour fundus photographs, Matsuba et al. proposed a CNN to classify between wet AMD and normal eye images; this CNN obtained a sensitivity of 100% and a specificity of 97% [31]. Keel et al. focused on the detection of the neovascular form of AMD by developing a DL approach based on a CNN (Inception-V3) for colour fundus images, which achieved a 97% sensitivity and a 96% specificity [32]. Treder et al. developed a program able to detect and classify geographic atrophy through a pre-trained CNN (Inception-V3) evaluating autofluorescence fundus photos; this CNN obtained a 100% sensitivity and a 92% specificity [33]. Burlina et al. trained a CNN (AlexNet) on over 130,000 colour fundus photographs, evaluating the absence or early stage vs. intermediate or late stage of AMD; this CNN reached a sensitivity of 85% and a specificity of 92% [34]. These performances seem superior to those obtained in the proposed fully automatic approach, combining the DL-based macular detector and the ML-based radiomic classifier, for which 93% sensitivity and 74% specificity were observed. However, it is notable that the aforementioned works differ from the present one for the definition of the target classes. In fact, the present work is the only one to include early stages of AMD in the positive class, aiming to detect early AMD in subjects. Thus, the proposed classification task is more challenging for anticipating treatment. On the other hand, the works from Matsuba et al. and Treder et al. included wet AMD cases in the positive class and healthy eye cases in the negative class, while Keel et al. and Burlina et al. included early-stage AMD in the negative class along with healthy subjects. In conclusion, the proposed classification task is similar but with different population cohorts, and performances cannot be properly compared.

As presented in the Results section, with our system, the human-assisted approach outperformed the fully automatic approach in terms of specificity, while the sensitivity maintained high values for both approaches. The decrease can be attributed to the small sample size of the training set for the macular detector, which, being based on DL techniques, is expected to require a large training set to reach its full potential. However, the clinical value of the fully automatic approach is promising, since the sensitivity has the greater impact in this clinical context. Ultimately, the proposed tool effectiveness should be evaluated in a real clinical setting, as a tool supporting the physician’s decision-making process, and in comparison with the clinical decision without the use of the tool. However, this was outside the scope of this work, the main objective of which was the evaluation of the potential of applying radiomics in UWF-FRT in an AMD context.

It should be remarked that CNNs usually guarantee good performances in classification tasks when a large number of images are available. On the other hand, the DL approach has a major flaw, lacking explainability, as discussed in the Introduction section, with this being the features learned by the model itself, which are very often unknown to clinicians. On the contrary, the proposed method, being a system of support vector machines based on a selection of IBSI-compliant radiomic predictors extracted from a standard macula region, presents a unique advantage for explainability. Radiomic predictors, able to discriminate eyes with and without AMD from quantitative texture features, represent crucial markers in terms of interpretation of the model to clinicians, consistently with elements investigated by the clinicians in the task of detecting AMD. First, the general meaning of the relevant radiomic predictors is addressed:

1. NGTDM Strength: quantifies the strength of the texture; the greater the strength, the easier it is to identify and clearly see the primitives of the texture, i.e., its fundamental patterns, structures, and elements. This feature can take high values in case of large primitives (even if low-contrasted) and in case of highly-contrasted primitives (even if small) [35].
2. NGTDM Contrast: quantifies how quickly the intensity values change across adjacent regions; the greater the contrast, the less smooth is the texture.
3. NGLDM Low Grey Level Count Emphasis: quantifies the degree at which large dark regions are present in the image; high values of this feature indicate greater presence of such regions.
4. NGLDM High Dependence Low Grey Level Emphasis: quantifies the dependence of dark regions; high values indicate that dark pixels tend to be in the same neighbourhoods.
5. GLRLM Long Run Low Grey Level Emphasis: quantifies the abundance of dark and long linear structures; the darker and/or the longer the structure, the greater the contribution to this feature is.
6. GLRLM Low Grey Level Run Emphasis: quantifies the abundance of linear structures, regardless of their length; the higher the value, the more linear structures are present in the image.
7. GLDZM Small Distance High Grey Level Emphasis: quantifies the presence of bright regions with small distance from the ROI’s borders; very bright and very peripheral regions contribute to high values of this feature.
8. GLRLM Short Run Low Grey Level Emphasis: similar to GLRLM Long Run Low Grey Level Emphasis, but small dark regions are emphasised.
9. NGLDM High Grey Level Count Emphasis: similar to NGLDM Low Grey Level Count Emphasis, but large bright regions are emphasised.
10. GLCM Joint Average: quantifies the smoothness of the regions in the image; high values indicate the presence of coarse regions.

The greater Strength (1), lower Contrast (2), and lower Joint Average (10) identified in eyes with AMD by the proposed SVM model is consistent with the presence of clearly detectable homogenous regions (e.g., drusen [33]). The features that emphasise the presence of dark structures (Low Grey Level Count Emphasis (3), High Dependence Low Grey Level Emphasis (4), Long Run Low Grey Level Emphasis (5), Low Grey Level Run Emphasis (6), Short Run Low Grey Level Emphasis (8)) show higher values in eyes with AMD, which is consistent with the presence of scattered dark areas (e.g., pigmentary abnormalities [36] and clearly visible macula). On the other hand, the two features that emphasise small or large bright regions (Small Distance High Grey Level Emphasis (7), High Grey Level Count Emphasis (9)) tend to be higher in eyes without AMD, as measured by our SVM model and consistent with the previous finding.

It could be questioned whether the Radiomic-based Machine Learning method is an appropriate processing method for analysing FRT. Our ML-based radiomics system shows good potential to become a useful explainable device applied to UWF-FRT, supporting clinical decision-making with high explainability and interpretability of mechanism of action. These results are the first, to our knowledge, that have been provided by a radiomic approach for AMD and applied to UWF-FRT, supporting the suitability of such feature extraction method combined with ML for the clinical use with this imaging modality. In addition to the results obtained, the work published by Liang et al. in 2021 [37] uses standard fundus photographs to predict diabetic foot, applying conventional radiomics approach combined with a SVM classifier. In such work, univariate analysis on the extracted features showed that some of the features from GLSZM and NGTDM families were significantly correlated with the class label (Diabetic Foot vs. Diabetes Mellitus). The final model, built on these features, proved to be able to discriminate between the two classes, with an overall AUC of 0.95 on the testing set.

It should be mentioned that the radiomic-based ML method was originally conceived for grey-level radiology images. However, FRT is able to detect the main specific retinal signs of degenerations, such as the presence of drusen, neovascularisations, fibrovascular scars, and intraretinal or subretinal macular fluids, that do not need colour information to be identified by the clinician. Thus, the proposed method is expected to be beneficial also for grey-level FRT as shown in the present work. Furthermore, texture analysis was well validated for RGB images, containing colour information, such as biomedical optical images in the optical domain. Radiomics-based approaches are expected to be properly applied also to RGB images such as colour-based FRT by extracting features from each colour channel [38].

Moreover, it is debatable if the proposed method, based on UWF-FRT, could be applied to standard FRT, too, since standard FRT contain the macula and the optic nerve [39]. Our method is based on two components: a DL-based macular detector and a ML-based radiomics model. The first component is expected to adapt very well to the standard FRT, since it has already learned all the primitives contained in standard FRT being trained on a wider field of view. However, since standard FRT and UWF-FRT can be translated to each other with DL methods, such as Generative Adversarial Networks [40], it could be possible, in principle, to develop a system able to generate standard FRT from the dataset used in this work, to be used with a fine-tuning approach; the DL-based macular detector may benefit from a few training epochs in order to specialise on such images. The second component of our method is based on a general approach that extracts the features just from the ROI, while ignoring the surrounding regions. Therefore, under the assumption that the ROI containing the macula is fully included in the standard FRT, the proposed system is expected to be able to capture image heterogeneity in this imaging modality, too.

## 5. Conclusions

In this work, a fully automatic ML classification tool was trained, validated, and tested, with the advantages of being highly explainable and interpretable by clinicians. The workflow developed included a combination of DL techniques, radiomics, and SVM techniques applied to UWF-FRT in patients at risk of AMD. Ultimately, the proposed system leverages an automatic detector to segment the macular region on UWF-FRT images, and a ML model to classify such region between any stage of AMD and healthy eyes. The obtained performances are promising and, once the effectiveness could be validated with a larger cohort, this tool may represent a valid support to physicians for decision-making in early AMD patients.

## Figures and Tables

**Table 1:** (**A**). Random forest classifiers|Classification performance: AUC, Accuracy, Sensitivity, Specificity, PPV, NPV, corresponding 95% CI, and statistical significance with respect to chance/random classification. Performance is reported for training, validation, and internal testing. (**B**). Support vector machine classifiers|Classification performance: AUC, Accuracy, Sensitivity, Specificity, PPV, NPV, corresponding 95% CI, and statistical significance with respect to chance/random classification. Performance is reported for training, validation, and internal testing.

	Training Set	Validation Set	Internal Testing Set
(**A**)			
ROC-AUC (%) (95% CI)	100 ** (99–100)	83 ** (82–83)	84 ** (83–84)
Accuracy (%) (95% CI)	100 ** (99–100)	78 ** (78–78)	81 ** (78–85)
Sensitivity (%) (95% CI)	100 ** (99–100)	71 ** (68–75)	72 ** (70–74)
Specificity (%) (95% CI)	100 ** (99–100)	83 ** (80–86)	88 ** (83–93)
PPV (%) (95% CI)	100 ** (99–100)	78 ** (77–79)	82 ** (75–89)
NPV (%) (95% CI)	100 ** (99–100)	81 ** (79–82)	81 ** (79–83)
(**B**)			
ROC-AUC (%) (95% CI)	96 ** (95–97)	90 ** (89–92)	87 ** (84–90)
Accuracy (%) (95% CI)	91 ** (88–93)	83 ** (82–85)	83 ** (79–86)
Sensitivity (%) (95% CI)	88 ** (85–91)	80 ** (78–82)	77 ** (69–87)
Specificity (%) (95% CI)	93 ** (90–95)	86 ** (85–87)	87 ** (85–89)
PPV (%) (95% CI)	92 ** (89–94)	83 ** (83–83)	82 ** (81–83)
NPV (%) (95% CI)	90 ** (87–92)	86 ** (85–88)	84 ** (78–89)

** *p*-value < 0.005.

**Table 2:** Support vector machine classifiers|Classification performance: Sensitivity, Specificity, PPV, NPV, corresponding 95% CI, and statistical significance with respect to chance/random classification. Performance is reported for the external testing dataset with images both manually and automatically segmented.

	Human Operator’s Manual ROI (Variable Radius)	DL-Based Macular Detector (250 Pixels Fixed Radius)
Sensitivity (%) (95% CI)	95.4 * (89.5–98.5)	92.6 * (85.9–96.7)
Specificity (%) (95% CI)	95.3 (84.2–99.4)	74.4 ** (58.8–86.5)
PPV (%) (95% CI)	98.1 (93.3–99.8)	90.1 ** (83.0–94.9)
NPV (%) (95% CI)	89.1 (76.4–96.4)	80.0 * (64.4–90.9)

* *p*-value < 0.05/** *p*-value < 0.005.

**Table 3:** Ensemble of support vector machine classifiers. The 10 top predictors are sorted in descending order according to their statistical significance and relevance.

Rank	Feature Family	Feature Nomenclature	Median in the AMD Class (95% CI)	Median in the Negative Class (95% CI)	Uncorrected *p*-Value	Corrected *p*-Value
1	Neighbourhood Grey Tone Difference Matrix	Strength	1.14 (0.97–1.31)	0.63 (0.57–0.69)	<0.005	<0.005
2	Neighbourhood Grey Tone Difference Matrix	Contrast	4.08 × 10^−2^ (3.43 × 10^−2^–4.72 × 10^−2^)	6.60 × 10^−2^ (5.62 × 10^−2^–7.59 × 10^−2^)	<0.005	<0.005
3	Neighbouring Grey Level Dependence Matrix	Low Grey Level Count Emphasis	3.08 × 10^−3^ (2.60 × 10^−3^–3.56 × 10^−3^)	2.17 × 10^−3^ (1.90 × 10^−3^–2.45 × 10^−3^)	<0.005	<0.05
4	Neighbouring Grey Level Dependence Matrix	High Dependence Low Grey Level Emphasis	3.91 × 10^−2^ (2.88 × 10^−2^–4.95 × 10^−2^)	2.30 × 10^−2^ (1.93 × 10^−2^–2.68 × 10^−2^)	<0.005	<0.05
5	Grey-Level Run Length Matrix	Long Run Low Grey Level Emphasis	7.75 × 10^−3^ (5.90 × 10^−3^–9.60 × 10^−3^)	4.75 × 10^−3^ (4.01 × 10^−3^–5.49 × 10^−3^)	<0.005	<0.05
6	Grey-Level Run Length Matrix	Low Grey Level Run Emphasis	3.05 × 10^−3^ (2.60 × 10^−3^–3.51 × 10^−3^)	2.18 × 10^−3^ (1.90 × 10^−3^–2.46 × 10^−3^)	<0.005	<0.05
7	Grey-Level Distance Zone Matrix	Small Distance High Grey Level Emphasis	36.59 (31.9–41.28)	48.98 (45.88–52.07)	<0.005	<0.05
8	Grey-Level Run Length Matrix	Short Run Low Grey Level Emphasis	2.57 × 10^−3^ (2.26 × 10^−3^–2.88 × 10^−3^)	1.81 × 10^−3^ (1.60 × 10^−3^–2.03 × 10^−3^)	<0.005	<0.05
9	Neighbouring Grey Level Dependence Matrix	High Grey Level Count Emphasis	684.57 (613.66–755.47)	910.92 (839.18–982.66)	<0.005	<0.05
10	Grey-Level Co-Occurrence Matrix	Joint Average	24.35 (23.02–25.67)	28.73 (27.48–29.97)	<0.005	5.72 × 10^−2^

## Data Availability

All data analysed for this study are presented in the article.
